# Recycled Aggregates from Construction and Demolition Waste in the Manufacture of Urban Pavements

**DOI:** 10.3390/ma14216605

**Published:** 2021-11-02

**Authors:** Manuel Contreras-Llanes, Maximina Romero, Manuel Jesús Gázquez, Juan Pedro Bolívar

**Affiliations:** 1 Research Centre for Natural Resources, Health and Environment (RENSMA), Department of Sociology, Social Work and Public Health, University of Huelva, 21007 Huelva, Spain; 2 Research Centre for Natural Resources, Health and Environment (RENSMA), Department of Integrated Sciences, University of Huelva, 21007 Huelva, Spain; bolivar@uhu.es; 3 Department of Materials, Instituto de Ciencias de la Construcción Eduardo Torroja (IETcc-CSIC), 28033 Madrid, Spain; nromero@ietcc.csic.es; 4 Department of Applied Physics, Marine Research Institute (INMAR), University of Cádiz, 11510 Cádiz, Spain; manueljesus.gazquez@uca.es

**Keywords:** recycled mixed aggregates, construction and demolition waste, treatments improvement, eco-efficient materials, urban pavements, environmental impact

## Abstract

Construction and Demolition Waste (CDW) is among the largest waste streams in the world. Therefore, within the Circular Economy concept, there is a growing interest in its reuse. The purpose of this work was to study the use of recycled aggregates (RAs) obtained by a specific separation method from CDW, replacing natural aggregates (NAs) in the manufacture of precast concrete elements, such as kerbstones and paver blocks. The physical and technological properties of precast products formulated with RAs were analysed in accordance with current regulations, comparing them with those of commercial products manufactured with NAs. The results indicated that partial or total substitution of NAs by RAs increased the water absorption and apparent porosity values of the precast elements while reducing the bulk density and compressive strength. However, all units manufactured with RAs showed breaking load values higher than the minimum required by EN 1338 and, in some cases, slightly higher average tensile strength values than the reference material. In addition, some of the compositions including RAs gave rise to pieces that, according to their flexural strength, were classified as class 1 and marked S in accordance with EN 1340. According to abrasion resistance, in most cases, the precast elements are classified as Class 4 and I (≤20 mm). Finally, precast concrete produced from RAs satisfies the tolerance requirements for classification as class 3 (≤1.5 kg m^−2^). Therefore, it could be suitable for use in high pedestrian or traffic areas.

## 1. Introduction

One of the strategic lines of the European Union policies aims to ensure the “Circular Economy” through the efficient use of raw materials and residues and the generation of clean energy. For that, policies on waste management must be directed in respect of the hierarchy of 4Rs established by the normative: reduction, reuse, recycle, and recovery [1].

In this sense, one of the most generated wastes at the global level is Construction and Demolition Waste (CDW). It is important to note that from mid-2008 until early 2013, with the economic and financial crisis, the construction sector was gradually reducing the consumption of raw materials and, therefore, the generation of wastes. Then, since the end of 2014, the construction industry began its recovery [2]. CDW is related to the wastes generally coming from the demolition, construction, and renovation activities of buildings, roads, and bridges, including inert, non-inert, hazardous, and non-hazardous materials as different as cement, concrete, wood, bricks, glass, ceramic, solvents, plastics, metals, and excavated materials (soil, gravel, rock, clay, and vegetation), among others [3,4].

In the EU, the construction sector represents 9% of the Gross Domestic Product (GDP) [5], generating approximately 374 million tonnes of CDW in 2016 (where the inert waste represents 90% of the total). This quantity represents the most important waste by weight [4]. In the USA, the percentage of the GDP from the construction sector is slightly lower at 4.3%, generating around 600 million tonnes of CDW in 2018 [6] (where 94.5% was generated during demolition activities and 5.5% during construction). In China, 1.15 billion tonnes were generated in 2014 [7], increasing to 2.64 billion tonnes in 2015, representing 30–40% of the total generated waste [8].

For that reason, there is an increasing interest at the global level of reuse and recycling of these CDW. RAs from CDW have usually been focused on concrete road and pavement applications. Thus, it has been studied at the laboratory scale in pavements [9]; the manufacture of precast concrete blocks made with recycled mixed ceramic aggregates from CDW [10]; the feasibility of sustainable construction materials for concrete paving blocks [11]; concrete kerbs and floor blocks with RAs [12], concrete pavement flags [13]; unbound layers of pavements [14]; concrete paving blocks and flags made with crushed brick as aggregate [15]; concrete paving block production [16]; the transportation geotechnics sub-base evaluating the crushing characteristics of CDW [17], road base and sub-base [18]; CDW with Portland cement and hydrated lime as pavement subbase [19]; paving stone, kerb, and concrete pipes [20,21]; and the manufacture of geopolymeric concrete units [22,23,24]. In a previous study, the authors highlighted the importance of Priot; to use it is necessary to carry out a separation process of the CDW in order to ensure that the RAs have the necessary quality as substitutes of NAs, i.e., improvement of the properties of RAs through different mechanical treatments. The main objective of this work was to study the use of RAs obtained by a specific separation method from CDW, analysing their final separation into fine (<4.8 mm) and coarse (>4.8 mm) aggregates with the aim to substitute the NAs, sand, and gravel in the manufacture of kerbstones and paving blocks, since previous work on the use of CDW as a replacement for NAs in the manufacture of paving blocks provided unsatisfactory results [15]. Subsequently, the physical and technological properties were analysed according to the current regulations, comparing these new samples with the commercial products manufactured with NAs. Therefore, the philosophy of the circular economy allows us to substitute traditional materials by construction and demolition wastes.

## 2. Materials and Methods

### 2.1. Materials and Sample Preparation

In the present study, 4 different aggregate types, natural sand (0–4 mm), natural gravel (4–12 mm), recycled sand (0–4 mm), and recycled gravel (4–12 mm) were used in the production of concrete specimens. Natural sand (NS) and gravel (NG) were supplied by the precast concrete company Montalbán y Rodríguez S.L., located in the region of Murcia (Spain). Recycled sand (RS) and gravel (RG) were provided by an integrated CDW treatment plant of the Murcia region. These types of RAs with physical and mechanical properties similar to those of NAs used in the present study are quite easy and cheap to obtain following the separation method reported in previous studies [25]. This methodology (crushing, grinding, sieving, and removal of impurities) eliminates the most fragile materials, concentrating those that are more resistant and suitable to be applied in concrete manufacture. On the other hand, Ordinary Portland cement (OPC) and superplasticiser additive were used in the preparation of concrete. This OPC Type I is characterised by a compressive strength of 32.5 N mm^−2^ and is composed of a mixture of clinker (97 wt.%) and natural gypsum (3 wt.%). Superplasticiser MasterCast 731, an additive-based polymer (polycarboxylate) supplied by BASF Company (Ludwigshafen, Germany), was used. According to the technical data, this additive can reduce the water content while maintaining workability and improving the strength, durability, and shrinkage of the concrete. Finally, potable water was used in the present research for both casting and curing of the concrete. Furthermore, the recycled aggregates (RS and RG) were pre-saturated for 3 min through soaking in potable water to avoid the high WA values compared to natural aggregates (NS and NG) due to the presence of porous materials, such as bonded mortar, ceramic, clay, etc. [26,27,28]. According to similar studies [25,29,30,31] the partial saturation (around 50% of the complete saturation) of the superficial pores guaranteed an appropriate consistency and workability with a minimum loss in the resistance of the final concrete. Additionally, this method reduces water absorption during the cementation process, keeping the process water-free until the cement hydration [10,30,31]. Consequently, pre-saturation is a suitable method to solve the problem of the higher porosity of recycled aggregates and has been used in this research.

**Figure 1:**
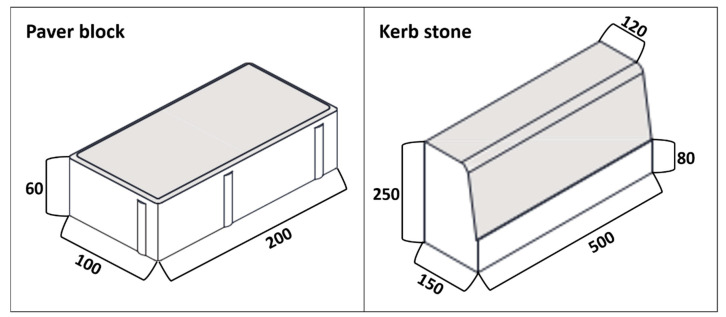
General appearance and dimensions (mm) of the paving blocks (**left**) and the kerbstone (**right**).

Concrete mixtures were formed to determine the physical and mechanical properties of the concrete specimens using these four types of aggregate, cement, water, and superplasticiser. Concrete mixtures were prepared by substituting recycled (0, 25, 50, and 100 wt.%) for natural aggregates. The aggregates (natural and/or recycled), cement, superplasticiser, and water percentage by weight of the concrete mixture are presented in Table 1. Preparation of all mixes was performed using the same technique and equipment. The mixes were homogenised and moistened by spraying water and superplasticiser using the optimum index supplied by the precast company for obtaining the best consistency and workability. All the mixtures were prepared using an effective water/cement ratio of 0.45 and 0.008% of additive (superplasticiser) over the weight of cement. According to the EN 12390-4 standard [32], cylindrical test specimens (Ø = 150 mm, h = 300 mm) were conformed, vibrated, and stored in open air with a temperature of 22–30 °C and a relative humidity of 65–75%. Additionally, paving blocks and kerb units were manufactured by pressing in duplicate, utilising a uniaxial hydraulic press at 30 tonnes in steel moulds, in accordance with industrial requirements (Figure 1).

### 2.2. Characterisation Techniques

The mineralogical composition of the raw materials was carried out using the XRD (X-ray diffraction) technique in a Shimadzu (Kyoto, Japan) diffractometer model XRD 6000 with Cu-Kα radiation and operating at 1.2 kW (40 kV e 30 mA). The diffractograms were registered in the interval of 5–60° 2θ, with a step size of 1° min^−1^. Moreover, the chemical analysis was performed using the energy dispersive X-ray fluorescence (EDXRF) technique in a Bruker (Billerica, MA, United States) S2 Ranger LE spectrometer fitted with a 50 W X-ray tube (50 kV, 2 mA), Pd anode, XFlash silicon drift detector with <135 eV resolution for Mn-Kα and 100.000 cps and equipped with a Peltier cooling system (liquid nitrogen is not required), and primary filter tool changers with nine positions possible. Finally, the trace elements were measured by inductively coupled plasma mass spectrometry (ICP-MS) by using HP computer model HP4500 (Palo Alto, CA, United States). The equipment was pre-calibrated with suitable standards.

In order to determine the physical properties of aggregates for concrete, bulk density (BD), specific gravity (SG), water absorption (WA), and Los Angeles (LA) abrasion loss were measured in accordance with the requirement established in the EN 12620 standard [33]. BD and WA were determined in standard procedures in accordance with the EN 1097-6 standard [34]. Additionally, the BD of aggregates was studied under two conditions: loose and packed. Loose conditions are dry materials that have been moved or agitated to loosen the natural packaging process, whereas packed conditions materials have been packed manually or mechanically (compacted). For measuring specific gravity (SG), samples were finely ground (< 62 µm). A weighed mass from this powder was used to determine its true volume and, therefore, its true density by displacing distilled water inside a pycnometer [35]. Determination of resistance to fragmentation (LA) was performed according the EN 1097-2 standard [36]. The standard LA abrasion test subjects a coarse aggregate sample (retained on the No. 12 (1.70 mm) sieve) to abrasion, impact, and grinding in a rotating steel drum containing a specified number of steel spheres. Once the test is complete, the calculated mass of aggregate that has broken apart to smaller sizes is expressed as a percentage of the total mass of aggregate. Therefore, lower LA abrasion loss values indicate aggregates that are tougher and more resistant to abrasion [36].

In addition to SG and WA, apparent porosity (AP) and compression strength (σ) were studied for concrete test specimens [37,38,39]. The test for SG was conducted by following the same standard employed for aggregates [35]. Moreover, the WA values of the prepared test specimens were determined and were expressed as a ratio of the mass of the absorbed water of an immersed specimen to the oven dried mass of the same specimen. Similarly, AP was calculated as the ratio of the mass of the absorbed water of an immersed specimen to the bulk volume of the specimen. The compressive strength was determined using an EMIC (Shinagawa-ku, Tokyo, Japan) apparatus, model DL-2000 at 28 days of curing according to the requirements and test methods established in the EN 12390-4 [40] standard. The compressive strength was calculated by dividing the failure load by the loading area of the paving blocks.

Finally, WA and strength properties, tensile splitting (T), and bending (B) strengths were evaluated for concrete precast paving. The test for WA was conducted by following the same previously reported procedure and was expressed as the ratio of the mass of the absorbed water of an immersed specimen to the oven-dried mass. Tensile splitting strength tests with concrete block paving were conducted to measure the indirect T. They were conducted in accordance with the requirement of EN1338 [41] and by using the EMIC (Shinagawa-ku, Tokyo, Japan) testing machine at 28 days of curing. Furthermore, BD test was carried out to determine the flexural strength of hardened kerb units after 28 days with three-point loading according to the EN1340 standard [42]. The abrasion resistance is a usual quality parameter used for precast concrete products, such as pavers and kerbs. The test was carried out by abrading the top face of both materials with an abrasive material under standard conditions, according to references [41,42] respectively. The freeze–thaw resistance was evaluated in 28-day cured concrete [41,42]. Each specimen was covered with a 5 mm thick layer of freezing medium (3 wt.% NaCl) and a plastic film with the aim of avoiding changes in the experimental conditions (evaporation and concentration). Then, the samples were introduced into a freezing chamber with temperatures ranging from −18 to 20 °C. During the thawing phase of the 7th, 14th, 21st, and 28th cycles, each specimen was rinsed with water into a filter paper to collect the scaled material, which afterwards was dried at 105 °C for at least 24 h. Freeze–thaw resistance was evaluated by measuring mass loss per unit area.

## 3. Results and Discussion

### 3.1. Raw Materials Characterisation

The chemical and mineralogical composition of the raw materials used in this study has been fully discussed in a previous paper [25]. Briefly, the major constituent element of recycled aggregates is silicon (78.4 and 73.0 wt.% SiO_2_ in RG and RS, respectively). In addition, their chemical composition includes minor components, such as aluminum (5.4–6.8 wt.% Al_2_O_3_), calcium (5.5–6.4 wt.% CaO), iron (1.9–2.3 wt.% Fe_2_O_3_), and magnesium (1.2–1.4 wt.% MgO), among others. In terms of mineralogy, the predominant crystalline phase in the recycled aggregates is quartz (SiO_2_), with calcite (CaCO_3_) and portlandite (Ca(OH)_2_) as secondary phases. Silicon oxide is also the major component of natural aggregates (90.6 wt.% SiO_2_ in NS and 63.2 wt.% SiO_2_ in NG), together with other minor elements, such as aluminum (3.8 wt.% Al_2_O_3_), magnesium (1.3 wt.% MgO), calcium (1.5 wt.% CaO), and iron (1.2 wt.% Fe_2_O_3_) in NS or calcium (10.5 wt.% CaO), aluminum (4.8 wt.% Al_2_O_3_), iron (2.9 wt.% wt.% Fe_2_O_3_), sodium (1.5 wt.% Na_2_O), and magnesium (1.2 wt.% MgO) in NG. As expected, quartz is also the principal crystalline phase found in natural aggregates, along with smaller amounts of calcite and feldspar in the case of NS. On the other hand, the chemical composition of Portland cement type I is mainly composed of CaO (59.9 wt.%) and SiO_2_ (21.5 wt.%), with Al_2_O_3_ (5.9 wt.%), Fe_2_O_3_ (2.8 wt.%), and MgO (2.2 wt.%) as minor components.

The physical and mechanical characteristics (BD, WA, SG, and LA) of natural and recycled aggregates are collected in Table 2. The bulk density (BD) was analysed under two different situations: loose and compacted condition. Both bulk density results follow a similar trend. The BD under loose condition of NS and NG are in the order of a magnitude of ∼1500 kg m^−3^, whereas the RS and RG are slightly lower, ~1450 kg m^−3^, by considering the experimental uncertainties, regardless of the type of CDW (∼1400 kg m^−3^). Furthermore, the results of BD obtained under compacted conditions of natural constituents (NS and NG) and recycled aggregates (RS and RG) were similar and around 1600 kg m^−3^. Similarly, the specific gravity (SG) was slightly higher in the natural aggregates (NS and NG, 2450 and 2650 kg m^−3^, respectively) in comparison to recycled aggregates (RS and RG, 2280 and 2390 kg m^−3^, respectively) because the density of natural constituents is higher than that of recycled aggregates [10,16,25]. Moreover, the recycled aggregates, RS and RG, met the requirements established in the EN 12620 standard [33], which specifies the required properties of natural, mechanically processed, recycled, or mixtures of aggregates to be used in concrete manufacture and requires aggregates with SG greater than 2000 kg m^−3^ (Table 2). On the other hand, the water absorption (WA) of CDW was 7.1% higher than recycled aggregates, 5.0% RS and 4.5% RG, and even higher than NS and NG, 1.1 and 1.7%, respectively. These higher WA values of RS and RG compared to NS and NG could be attributed to their lower density and consequently higher porosity. Furthermore, by comparing the WA results with the WA limits (below 5.0%) recommended in the structural concrete instruction (EHE-08) approved by the Royal Decree 1247/2008 of July 18 [43], it can be said that both recycled aggregates met this recommendation. In light of the above, the presence of porous materials, such as ceramics and bonded mortars, included in the recycled aggregates (RS and RG) produced values for both SG and bulk density (loose and compacted) which were lower, and WA results which were higher compared to NAs.

Finally, the mechanical properties of coarse aggregates (NG and RG) were studied according to Los Angeles abrasion (LA) test [36]. The EHE-08 stated that the limit value of the Los Angeles abrasion loss is 40% with a maximum of 500 revolutions [43]. Moreover, an LA abrasion loss value of 40 indicates that 40% of the original sample passed through the No. 12 (1.70 mm) sieve. Therefore, lower LA abrasion loss values indicate aggregates that are tougher and more resistant to abrasion. The results revealed that both NG and RG were within the established limits, 25 and 33%, respectively (Table 2). The presence of bonded mortar, ceramic materials, etc. in the RG may have been responsible for the high abrasion values compared to NG.

### 3.2. Concrete Test Characterisation

Table 3 shows several technological properties, such as water absorption (WA), apparent porosity (AP), real density (SG), and compressive strength (σ) determined in concrete test specimens manufactured with different substitutions of NAs by RAs. Similar results were obtained in other studies [44,45,46,47]. It is noted that, in general, the addition of RAs increases the WA and AP of concrete test specimens while reducing the SG and σ values.

The addition of recycled aggregates, either RS or RG, to replace natural aggregates results in a slight increase in water absorption values (Table 3). Even so, concrete manufactured with up to 75 wt.% recycled aggregates replacement (RS or RG) present water absorption values below 6%. On the other hand, concrete elements manufactured with simultaneous substitution of RS and RG recycled aggregates are those that present a greater increase in water absorption. In relation to the porosity at 28 days (Table 3), the value recorded for the control precast (0 wt.% recycled aggregate) was slightly lower (12.37%) than that of the one containing 25 wt.% of RAs (12.44%), since the volume of pores is higher in the concrete elements manufactured with recycled aggregates. The values fluctuate between 12.44% and 21.5% for those containing 100 wt.% substitution. Concerning the density values (Table 3), the replacement of both the sand and gravel fractions of natural aggregates by recycled aggregates results in a decrease in the density and therefore an increase in the volume of the pieces. This increment in volume must be associated with an increase in internal porosity, which is in accordance with the aforementioned changes in porosity and water absorption [10,44,45]. In general, in concrete test specimens prepared with RAs, the absolute error of the AP and WA measurements decreases, and, therefore, the repeatability improves, with this improvement being more considerable as the percentage of replacement of NAs by RAs increases. In addition, the repeatability is not affected in the case of density measurements, as the error remains practically unchanged.

**Figure 2:**
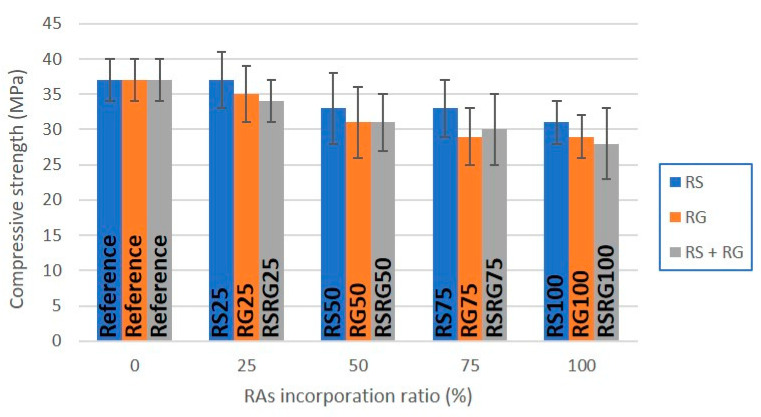
Characteristic compressive strength at 28 days for cylindric test specimens prepared with RAs.

Test specimens containing RAs show compressive strength values in the range 28–37 MPa, while the reference material reached an average value of 37 MPa (Table 3). Therefore, a decrease of approximately 0–24% in the compressive strength of precast concrete elements with recycled aggregates was observed (Figure 2). However, the replacement of 50 wt.% of the natural aggregate by RS resulted in very similar strength values, with a difference of less than 5% compared to the values obtained in precast concrete products prepared with natural aggregates. In contrast, lower strength values were obtained for replacements greater than 75 wt.%, achieving a 24% reduction in the compressive strength of materials obtained by complete replacement of NAs with RAs. The reduction in mechanical resistance is likely to be affected by the increase in porosity observed in the parts as the percentage of aggregate replacement increases. In fact, the total replacement of NAs by RAs is accompanied by an increase in porosity of 15 and 29%, respectively, while the compressive strength decreases by 17 and 22%, accordingly. In addition, the presence of bonded mortar, ceramic materials, etc., in the recycled aggregates may have been responsible for the observed loss of compressive strength [25,28,46,47]. In contrast to the former properties, in this case, the absolute error increases, and, therefore, the repeatability decreases for concrete specimens manufactured with RAs.

### 3.3. Paving Unit Characterisation

**Figure 3:**
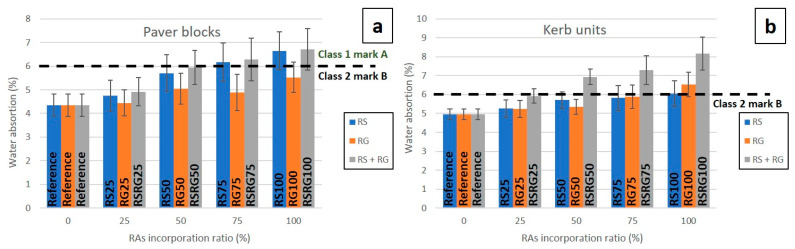
Graphical representation of water absorption vs. RAs’ incorporation ratio in pavers (**a**) and kerbs (**b**).

Once the RAs’ incorporation had been studied in concrete with satisfactory results, both the kerb units and the paver blocks containing RAs were prepared on a laboratory scale. Figure 3 shows the outcome of using RAs as a substitute for NAs on the water absorption of concrete pavers and kerbs. The partial or total inclusion of RAs increased the water absorption of the precast concrete elements. This behaviour has already been reported by other studies [20,48,49], and it is attributed to the high hygroscopicity of RAs, which presents a high ability to adsorb water in contrast to NAs, which exhibit lower permeability characteristics. In pavers blocks, the incorporation of RS has a more marked effect on the increase in water absorption than the incorporation of RG. Thus, total RS (RS100) or RS + RG (RSRG100) replacement resulted in a 53–55% increase in water absorption of the pavers, while this increase was 27% in the samples produced with only RG (RG100) substitution (Figure 3a). According to Nandi et al. [50], the higher absorption values of blocks prepared with RS substitution are due to the presence of a large amount of dust, which is a water absorber, adhering to their surface and increasing the water absorption of the pieces. In the case of kerbs (Figure 3b), the effect of the incorporation of RS compared to RG is not as noticeable, and the water absorption of the elements produced with RG is even slightly higher. This result is probably due to the different dosage of RS and RG in pavers and kerbs, since the proportion of RG in the latter is considerably higher. Thus, kerbs produced with 100 wt.% RG substitution (RG100) have an absorption value 32% higher than kerbs produced with natural aggregates (Reference), while the total replacement of RS increases the value by 22%. However, in the case of the kerbs, the joint substitution of RS and RG in the composition of the precast elements has the greatest detrimental effect, reaching a water absorption 65% higher than that of elements manufactured with NAs. Regarding the repeatability of the water absorption values, it slightly decreases for paver blocks as the percentage of substitution of RAs by NAs increases, this decrease being more considerable for kerb units. This behaviour is also observed in the analysis of other properties, such as apparent porosity, specific gravity, abrasion wear, bending strength, or tensile splitting strength.

**Figure 4:**
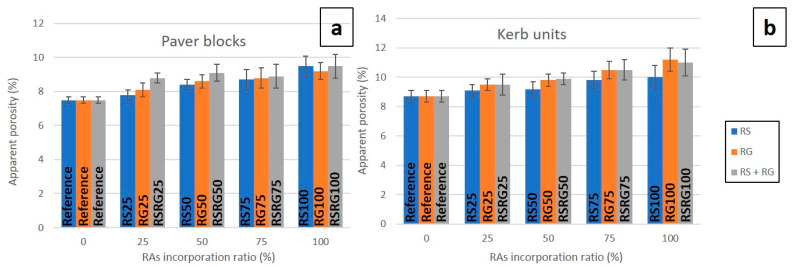
Graphical representation of apparent porosity vs. RAs’ incorporation ratio in pavers (**a**) and kerbs (**b**).

Regarding apparent porosity, Figure 4 depicts the influence on this property of partial or total replacement of NAs by RAs. As expected, similar to that observed for water absorption, apparent porosity increases with the percentage of natural aggregate that is replaced so that pavers manufactured with full substitution achieve porosity values approximately 25% higher than those from natural aggregates (Figure 4a). Concerning kerbs units (Figure 4b), the increase in apparent porosity is more dependent on RS (RS100), RG (RG100), and RS + RG (RSRG100) occurrence. However, in general, the effect of the inclusion of recycled aggregates on the increase in apparent porosity is lower than the corresponding effect on water absorption, indicating a prevalence of water absorption by hygroscopicity of the recycled aggregates as against capillary absorption through the pore system [47,49].

**Figure 5:**
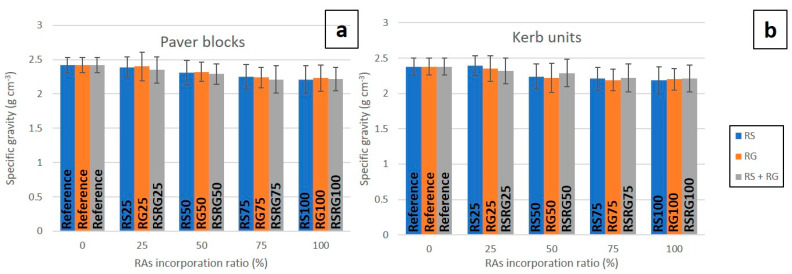
Graphical representation of specific gravity vs. RAs’ incorporation ratio in pavers (**a**) and kerbs (**b**).

Figure 5 presents the evolution of the specific gravity of cured pavers and kerbs with increasing substitution of natural aggregate by recycled aggregate. The use of the recycled aggregates resulted in precast concrete elements with a slightly lower specific gravity than those manufactured with natural aggregates. This decrease may be due to the lower density of RAs in relation to NAs due to the presence of adhered mortar, clay-based particles, and floating materials [51], resulting in lower density fresh masses [52].

**Figure 6:**
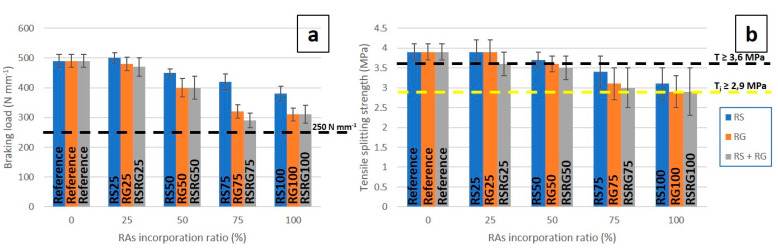
Graphical representation of breaking load (**a**) and tensile splitting strength (**b**) vs. RAs’ incorporation ratio in pavers.

Furthermore, in line with the results of previous research [50,53], the strength decreased to a varying level as a result of the RAs’ content. The decrease in strength values could be explained by a worse aggregate–cement bonding due to the presence of traces of other components of the CDW adhered to the RAs’ surface. Figure 6 illustrates the results of breaking load by unit of length and tensile strength determined for conventional concrete paver blocks and for those with recycled aggregates. In all cases, prefabricated elements prepared with recycled aggregates showed values of breaking load over the minimum of 250 N mm^−1^ stated in the EN-1338 [41] standard, achieving an average value of breaking load close to 500 N mm^−1^ (Figure 6a). Compared to concrete samples free of RAs, the total replacement of RS resulted in a concrete breaking load of about 22%, while the use of RG or a mixture of RS and RG reduced the concrete strength by up to 37%.

With regard to the tensile splitting strength, all the prefabricated elements exceeded the minimum value of 2.9 MPa for individual samples (Figure 6b). In addition, recycled pavers with up to 25 wt.% of recycled aggregates (RG and RS) showed an average tensile strength of 3.7 MPa, exceeding the threshold of 3.6 MPa set in the standard. Therefore, the comparison of the tensile strength of concrete pavers made from conventional and recycled aggregates showed that a 50 wt.% replacement of NAs with RAs does not have a significant effect on the mechanical performance of paver blocks. On the other hand, paver blocks prepared with recycled aggregates above 75 wt.% replacement did not exceed the tensile splitting strength threshold of 3.6 MPa. However, the study shows that replacements of up to 50 wt.% of RS (RS50) or RG (RG50), or 25 wt.% of RS + RG (RSRG25), fulfil the requirements for pavers.

Figure 6b shows that the tensile strength is mostly influenced by the characteristics of the interfacial transition zone between cement paste and aggregate [29,54]. In general, the incorporation of RAs reduces the tensile splitting strength, this reduction being more considerable as the percentage of NAs’ replacement by RAs increases so that a reduction of between 21 and 26% is achieved for the total substitution. This performance could be attributed to the existence of a porous interlayer within the aggregate and the cement body [30,55].

**Figure 7:**
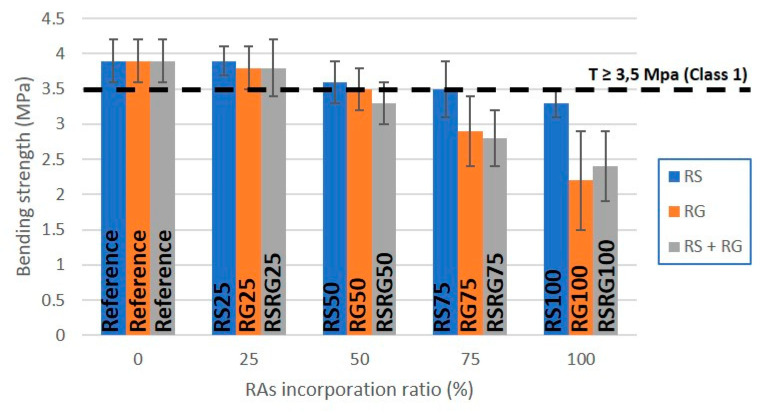
Graphical representation of bending strength vs. RAs’ incorporation ratio in kerbs.

The bending strength of concrete kerb units manufactured from recycled aggregates having different particle size is presented in Figure 7. As observed with the compressive strength, the bending strength decreases as the percentage of natural aggregate replacement increases, with the precast concrete products prepared with RS showing better performance. Similar results were observed in previous studies [11,13,14,21]. The materials manufactured with 100 wt.% of RS (RS100) or 75 wt.% of RG (RG75) or 50 wt.% of RS + RG (RSRG50) presented values of resistance to flexion below 3.5 MPa. Prefabs containing up to 75 wt.% RS (RS75) and samples with 50 wt.% RG (RG50) of replacement are located on the threshold class 1 and S mark, while materials prepared with less than 50 wt.% replacement show bending strength values greater than 3.5 MPa. According to the EN 1340 [42], these are classified as class 1 and S mark (characteristic bending strength ≥3.5 MPa; minimum bending strength ≥2.8 MPa), which means that they can be used in pedestrian areas or areas with little traffic.

When compared with the concrete samples manufactured with NAs, the overall replacement of RS resulted in a 15% reduction in flexural strength, the decrease being larger when RG is replaced so that a decrease of approximately 44% and 38% is achieved when full replacement of RG or RS + RG occurs, respectively (Figure 7).

**Figure 8:**
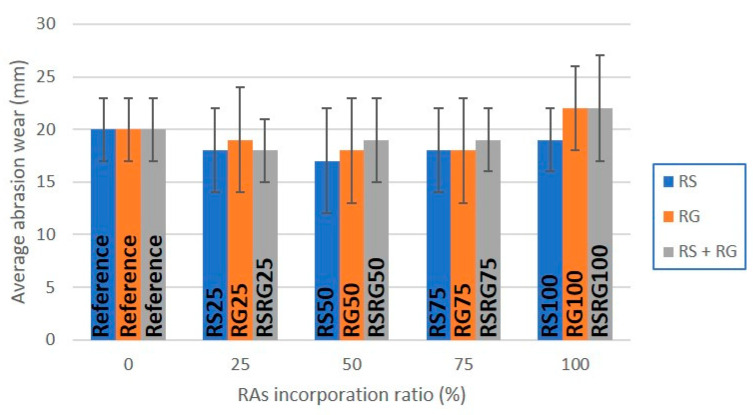
Graphical representation of the average abrasion wear vs. RAs’ incorporation ratio in pavers.

Figure 8 shows the abrasion loss suffered by the different precast concrete elements, which is one of the most usual indicators of durability for this type of element. The replacement of NS by RS resulted in an improvement of the abrasion resistance. In most cases, precast elements prepared with RAs showed values below the 20 mm displayed by the reference samples. The improved abrasion resistance is probably due to a higher density of the paste, as well as a stronger bond between the cement paste and the RAs. Moreover, the replacement of the RG and the simultaneous replacement of RS leads to a similar result, with the exception of the prefabricated elements manufactured with 100 wt.% replacement. The precast control elements (References) and those prepared with 100 wt.% replacement of either the RG (RG100) or the simultaneous replacement of RS and RG (RSRG100) are classified as Class 3 and H, since their average abrasion wear was above 20 mm and below 23 mm in accordance with the abrasion resistance described in Annexes G for kerbs [41] and paving stones [42] standards. However, the rest of precast concrete elements presented values of abrasion resistance within the tolerance limits to be classified as Class 4 and I (≤20 mm of abrasion wear). Therefore, these precast elements supplemented with RAs could be suitable for use in areas of very heavy pedestrian and vehicle traffic.

**Figure 9:**
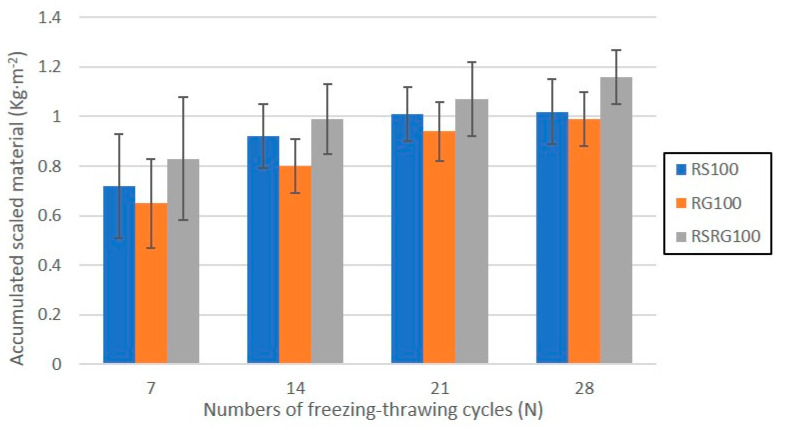
Graphical representation of the accumulated scaled material vs. numbers of freezing–thrawing cycles.

The progression of the accumulated scaled material of precast concrete samples prepared with 100 wt.% of RAs over 28 freeze–thaw cycles is illustrated in Figure 9. The overall loss of mass of the concrete elements after 28 freeze–thaw cycles was approx. 1.15 kg m^−2^. Accordingly, precast concrete produced from recycled aggregates satisfies the tolerance requirements for classification as class 3 in accordance with Annexes E [41,42]. Furthermore, the standard establishes that the loss of individual mass must be lower than 1.5 kg m^−2^. This requirement is satisfied by all concrete elements manufactured with the substitution of NAs by RAs, as in the most unfavourable situation (complete replacement of RS and RG and 28 freeze–thaw cycles) the loss of mass achieved the maximum value of 1.3 kg m^−2^. The control prefabricated units were also tested, exhibiting similar values to the precast elements with recycled aggregates. Unlike the previous properties, the repeatability of the freeze–thaw resistance values increases considerably in the concrete elements with substitution of NAs for RAs, with the increase being greater as the percentage of replacement increases.

## 4. Conclusions

In this manuscript, the use of RAs, fine (RS) and coarse (RG), obtained by a specific separation method from CDW as substitution of NAs, sand (NS) and gravel (NG), in the manufacture of kerbstone and paving blocks was analysed.

In relation to the physical properties, bulk density was quite similar for NAs (1500 kg m^−3^) and RAs (1450 kg m^−3^) in loose condition and 1600 kg m^−3^ in compacted conditions for both NAs and RAs samples. Specific gravity (SG) was slightly higher in the NAs (sand and gravel, 2450 and 2650 kg m^−3^, respectively) in comparison to RAs (RS and RG, 2280 and 2390 kg m^−3^, respectively). The mechanical properties of coarse aggregates (NG and RG) show a Los Angeles abrasion test value of 25 and 33%, respectively, within the limit given by current regulation.

Technological properties of concrete specimens obtained with different substitutions of NAs (NS and NG) by RAs (RS and RG) were carried out. As a general conclusion, the addition of RAs increases the water absorption and apparent porosity, reducing the real density and compressive strength. Specifically, samples with a substitution of NAs by RAs at 25 wt.% (RS25, RG25, and RSRG25) show a similar compressive strength (37 ± 4, 35 ± 4 and 34 ± 3 MPa) with a reference material (37 ± 3 MPa).

Finally, the kerbs and paver blocks were manufactured at laboratory scale in order to evaluate the breaking load and tensile splitting strength. All specimens manufactured with RAs showed values of breaking load higher than the minimum given by EN-1338, 250 Nmm^−3^, while a substitution of 25 wt.% of RAs (RG and RS) presented an average tensile strength of 3.7 MPa, slightly higher than a reference material (3.6 MPa). Additionally, replacements up to 50 wt.% of RS (RS50) or RG (RG50) or 25 wt.% of RS + RG (RSRG25) fulfil the requirements for pavers established in the EN-1338 standard. Moreover, bending strength of concrete kerbs manufactures samples was evaluated. The samples with replacements of 25 wt.% of NAs by RAs (RSRG25) are clearly higher than 3.5 MPa, being classified according to the EN 1340 as class 1 and S mark. According to abrasion resistance, most elements manufactured are classified as Class 4 and I (≤20 mm of abrasion wear) while reference RG100 and RSRG100 belong to Class 3 and H (≤23 mm). Finally, precast concrete elements produced with RAs satisfy the tolerance requirements for classification as class 3, for which the loss of individual mass must be lower than 1.5 kg m^−2^. Therefore, these precast elements can be used in pedestrian areas or areas with little traffic.

Regarding the repeatability of the values, in general, the absolute error increased, and the repeatability decreased in the units manufactured with RAs, with the exception of the freeze–thaw resistance where a considerable increase in repeatability is observed as the percentage of substitution of NAs by RAs increases.

## Figures and Tables

**Table 1:** Mix proportion (% by weight) of the different raw materials used in the test concrete specimens. Sample codes were labelled according the type (recycled sand [RS] and/or recycled gravel [RG]) and the percentage (25, 50, 75, and 100 wt.%) of each recycled aggregate added in the mixes. A reference sample was manufactured without recycled aggregates.

					Natural Aggregates		Recycled Aggregates	
Sample	Water Content	Cement Content	W/C Ratio	Superplasticiser	Gravel (NG)	Sand (NS)	Gravel * (RG)	Sand * (RS)
Reference	3.02	6.76	0.45	0.05	36.07	54.10	-	-
RS25	3.02	6.76	0.45	0.05	36.07	40.58	-	13.53
RS50	3.02	6.76	0.45	0.05	36.07	27.05	-	27.05
RS75	3.02	6.76	0.45	0.05	36.07	13.53	-	40.58
RS100	3.02	6.76	0.45	0.05	36.07	-	-	54.10
RG25	3.02	6.76	0.45	0.05	27.06	54.10	9.02	-
RG50	3.02	6.76	0.45	0.05	18.04	54.10	18.04	-
RG75	3.02	6.76	0.45	0.05	9.02	54.10	27.06	-
RG100	3.02	6.76	0.45	0.05	-	54.10	36.07	-
RSRG25	3.02	6.76	0.45	0.05	27.06	40.58	9.02	13.53
RSRG50	3.02	6.76	0.45	0.05	18.04	27.05	18.04	27.05
RSRG75	3.02	6.76	0.45	0.05	9.02	13.53	27.06	40.58
RSRG100	3.02	6.76	0.45	0.05	-	-	36.07	54.10

* Recycled aggregates were used under pre-saturation condition.

**Table 2:** Average mechanical and physical properties (n = 10) of aggregates. Uncertainties given as standard deviation of the mean: u = (S_x_/n^1/2^), S_x_ being the standard deviation of the samples.

	BD * (kg m^−3^)	BD ** (kg m^−3^)	SG (kg m^−3^)	WA (%)	LA (%)
NS	1450 ± 70	1660 ± 40	2450 ± 180	1.1 ± 0.2	-
NG	1520 ± 40	1620 ± 90	2650 ± 110	1.7 ± 0.5	25 ± 1
CDW	1390 ± 180	1610 ± 190	2200 ± 340	7.1 ± 1.9	-
RS	1410 ± 120	1590 ± 100	2280 ± 290	5.0 ± 0.9	-
RG	1490 ± 190	1530 ± 90	2390 ± 210	4.5 ± 1.1	33 ± 3

* Loose condition. ** Compacted condition.

**Table 3:** Technological properties (n = 10) of the different compositions of concrete test specimens prepared with RAs (after 28 days of curing). Uncertainties given as standard deviation of the mean: u = (S_x_/n^1/2^), S_x_ being the standard deviation of the samples.

	AP (%)	SG (g cm^−3^)	WA (%)	σ (MPa)
Reference	8.7 ± 0.7	2.39 ± 0.22	4.8 ± 0.8	37 ± 3
RS25	9.1 ± 0.4	2.28 ± 0.20	5.1 ± 0.5	37 ± 4
RS50	9.2 ± 0.5	2.19 ± 0.18	5.7 ± 0.9	33 ± 5
RS75	9.8 ± 0.6	2.17 ± 0.23	5.8 ± 0.4	33 ± 4
RS100	10.0 ± 0.5	2.17 ± 0.20	6.1 ± 0.7	31 ± 3
RG25	9.5 ± 0.4	2.32 ± 0.10	5.0 ± 0.5	35 ± 4
RG50	9.9 ± 0.4	2.28 ± 0.24	5.4 ± 0.7	31 ± 5
RG75	10.5 ± 0.6	2.17 ± 0.19	5.9 ± 0.4	29 ± 4
RG100	11.2 ± 0.5	2.14 ± 0.22	6.5 ± 0.6	29 ± 3
RSRG25	9.5 ± 0.7	2.37 ± 0.25	5.9 ± 0.4	34 ± 3
RSRG50	10.1 ± 0.4	2.24 ± 0.15	6.9 ± 0.6	31 ± 4
RSRG75	10.5 ± 0.7	2.16 ± 0.31	7.3 ± 0.4	30 ± 5
RSRG100	11.0 ± 0.5	2.21 ± 0.21	8.2 ± 0.3	28 ± 5

## Data Availability

Not applicable.
